# Role of the phosphopantetheinyltransferase enzyme, PswP, in the biosynthesis of antimicrobial secondary metabolites by *Serratia marcescens* Db10

**DOI:** 10.1099/mic.0.078576-0

**Published:** 2014-08

**Authors:** Amy J. Gerc, Nicola R. Stanley-Wall, Sarah J. Coulthurst

**Affiliations:** Division of Molecular Microbiology, College of Life Sciences, University of Dundee, Dundee DD1 5EH, UK

## Abstract

Phosphopantetheinyltransferase (PPTase) enzymes fulfil essential roles in primary and secondary metabolism in prokaryotes, archaea and eukaryotes. PPTase enzymes catalyse the essential modification of the carrier protein domain of fatty acid synthases, polyketide synthases (PKSs) and non-ribosomal peptide synthetases (NRPSs). In bacteria and fungi, NRPS and PKS enzymes are often responsible for the biosynthesis of secondary metabolites with clinically relevant properties; these secondary metabolites include a variety of antimicrobial peptides. We have previously shown that in the Gram-negative bacterium *Serratia marcescens* Db10, the PPTase enzyme PswP is essential for the biosynthesis of an NRPS-PKS dependent antibiotic called althiomycin. In this work we utilize bioinformatic analyses to classify PswP as belonging to the F/KES subfamily of Sfp type PPTases and to putatively identify additional NRPS substrates of PswP, in addition to the althiomycin NRPS-PKS, in *Ser. marcescens* Db10. We show that PswP is required for the production of three diffusible metabolites by this organism, each possessing antimicrobial activity against *Staphylococcus aureus*. Genetic analyses identify the three metabolites as althiomycin, serrawettin W2 and an as-yet-uncharacterized siderophore, which may be related to enterobactin. Our results highlight the use of an individual PPTase enzyme in multiple biosynthetic pathways, each contributing to the ability of *Ser. marcescens* to inhibit competitor bacteria by the production of antimicrobial secondary metabolites.

## Introduction

Phosphopantetheinyltransferase (PPTase) enzymes have essential roles in the biosynthetic pathways of primary and secondary metabolism in prokaryotes, archaea and eukaryotes (Beld *et al.*, 2014; Lambalot *et al.*, 1996). Fatty acid synthases (FASs), polyketide synthases (PKSs) and non-ribosomal peptide synthetases (NRPSs) are all dependent upon the catalytic action of PPTase enzymes to function (Beld *et al.*, 2014). FASs, PKSs and NRPSs are generally large, multimodular proteins. Each module within the protein incorporates one acyl or amino acid unit into the growing metabolite and comprises a minimum of three essential domains, including the carrier protein domain (Fischbach & Walsh, 2006). PPTase enzymes catalyse the transfer of a 4′-phosphopantetheine (PPT) prosthetic group from coenzyme A to a conserved serine residue in the carrier protein domain of FASs, PKSs and NRPSs (Beld *et al.*, 2014; Elovson & Vagelos, 1968; Lambalot *et al.*, 1996). The 4′-phosphopantetheine prosthetic group of the carrier protein domain fulfils two main functions: the thiol-terminated 4′-phosphopantetheine arm serves as the point of attachment for the FAS, PKS or NRPS intermediate to the biosynthetic machinery and the flexibility of this arm allows the biosynthetic intermediate access to the catalytic reaction centres within the FAS, PKS or NRPS module (Beld *et al.*, 2014).

PPTase enzymes can be divided into three families: the Sfp family whose prototype is Sfp of *Bacillus subtilis* (Lambalot *et al.*, 1996; Reuter *et al.*, 1999); the AcpS family whose prototype is AcpS of *Escherichia coli* (Elovson & Vagelos, 1968; Lambalot & Walsh, 1995); and a third family whose members are integrated as domains within the FAS or PKS enzyme that they modify (Beld *et al.*, 2014; Copp & Neilan, 2006; Lambalot *et al.*, 1996; Walsh *et al.*, 1997). The Sfp family of PPTases have broad substrate specificity and are generally required for the activation of PKS and NRPS enzymes of secondary metabolism (Mootz *et al.*, 2001; Quadri *et al.*, 1998); these enzymes are approximately 230 residues in length and exist as monomers (Reuter *et al.*, 1999). Based on sequence comparisons, members of the Sfp family were further divided into the F/KES and W/KEA subfamilies (Copp & Neilan, 2006; Lambalot *et al.*, 1996). F/KES subfamily members were shown to largely be associated with NRPS enzymes whereas W/KEA subfamily members were more often associated with PKS enzymes (Copp & Neilan, 2006). Members of the AcpS family of PPTases are generally around 120 residues in length and are thought to exist as trimers (Chirgadze *et al.*, 2000; Parris *et al.*, 2000). The AcpS family of PPTases show a higher degree of specificity towards the carrier protein domain that they modify and are generally required for the modification of FAS enzymes from primary metabolism (Lambalot *et al.*, 1996; Mootz *et al.*, 2001; Parris *et al.*, 2000). Despite sharing limited overall sequence similarity, the AcpS and Sfp family members share homology at the structural level; an AcpS dimer resembles one Sfp monomer (Lambalot *et al.*, 1996; Reuter *et al.*, 1999). As previously described, there exists a third family of PPTases that are translationally fused to the FAS or PKS enzyme that they modify (Copp & Neilan, 2006; Fichtlscherer *et al.*, 2000; Lambalot *et al.*, 1996). Examples of these integrated PPTases are few and, to date, only two bacterial integrated PPTases have been experimentally validated (Murugan & Liang, 2008; Weissman *et al.*, 2004).

*Serratia marcescens* is a Gram-negative bacterium belonging to the family *Enterobacteriaceae*. Members of this genus are known to produce a variety of secondary metabolites (Fineran *et al.*, 2005; Kadouri & Shanks, 2013; Thomson *et al.*, 2000). Many strains, including *Ser. marcescens* ATCC 274, produce the secondary metabolite prodigiosin that gives the bacterium a distinctive red colour (Williamson *et al.*, 2006). *Ser. marcescens* Db10 is a model insect pathogen but lacks the genes to produce prodigiosin and so is an example of a non-pigmented strain of *Serratia* (Flyg *et al.*, 1980). However, we recently described the production of the antibiotic secondary metabolite, althiomycin, by *Ser. marcescens* Db10 (Gerc *et al.*, 2012). Althiomycin is the product of the six-gene *alb* operon, which encodes the following: a hybrid NRPS-PKS enzyme, appropriate tailoring enzymes and an export/resistance protein. The PPTase encoded by *SMA2452*, which is not genetically linked with the *alb* operon, was shown to be required for althiomycin biosynthesis in this organism (Gerc *et al.*, 2012). PswP, in *Ser. marcescens* ATCC 274, is the PPTase required for the production of prodigiosin and the surfactant serrawettin W1 (Sunaga *et al.*, 2004). *Ser. marcescens* Db10 does not produce serrawettin W1 dependent on the NRPS SwrW (Li *et al.*, 2005), instead, it produces serrawettin W2 dependent on the NRPS SwrA (Matsuyama *et al.*, 1992). Serrawettins W1 and W2 are both cyclic lipopeptides but have quite distinct chemical structures (Matsuyama *et al.*, 1992; Wasserman *et al.* 1962). The PPTase SMA2452 and PswP from *Ser. marcescens* ATCC 274 show 96 % identity at the protein level; therefore, SMA2452 will henceforth be referred to as PswP.

In this study, we provide evidence that PswP is required for the biosynthesis of two further secondary metabolites in *Ser. marcescens* Db10 and, in addition to althiomycin, these metabolites have an antimicrobial effect against *Staphylococcus aureus*. The production of one of these secondary metabolites, serrawettin W2, is known to be dependent on the action of PswP (Pradel *et al.*, 2007). We further show that the second PswP-dependent metabolite is a siderophore, likely enterobactin or a related molecule. Together, althiomycin, serrawettin W2 and the siderophore comprise a repertoire of secondary metabolites that have antimicrobial activity and are dependent on PswP for their biosynthesis.

## Methods

### Strains and culture media.

Strains used in this study are detailed in Table 1. *E. coli* was routinely cultured in Luria–Bertani (LB) medium (1 %, w/v, Bacto tryptone, 0.5 %, w/v, Bacto yeast extract, 1 %, w/v, NaCl), *Ser. marcescens* in low salt Luria–Bertani (LB) medium (1 %, w/v, Bacto tryptone, 0.5 %, w/v, Bacto yeast extract, 0.5 %, w/v, NaCl) and *Sta. aureus* in tryptic soya broth (TSB) (0.5 %, w/v, NaCl, 0.5 %, w/v, soytone, 1.5 %, w/v, tryptone). Chrome azurol S (CAS) agar plates were prepared as described by Schwyn & Neilands (1987). Growth media were solidified through addition of agar to 1.5 % (w/v). When required, media were supplemented with FeCl_3_, to a final concentration of 50 μM, or with antibiotics: 100 µg ampicillin ml^−1^; 1000 µg kanamycin ml^−1^ and 100 µg streptomycin ml^−1^. 

**Table 1.** Bacterial strains and plasmids used in this study

Strain/plasmid	Description	Source or reference
**Strains**		
Db10	*Serratia marcescens* wild-type	Flyg *et al.* (1980)
*Staphylococcus aureus* 113	ATCC 35556	Professor T. Palmer (University of Dundee)
*Staphylococcus aureus*	Newman *spa* : : tet *sbi* : : kan	Sibbald *et al.* (2010)
JESM267	Db10 (*swrA* : : Tn*5*)	Pradel *et al.* (2007)
SAN5	Db10 (Δ*alb4-5*) in-frame	Gerc *et al.* (2012)
SAN112	Db10 (Δ*pswP* : : *cml*)	Gerc *et al.* (2012)
SAN124	Db10 (Δ*alb4-5*, *swrA* : : Tn*5*)	This study
SAN176	Db10 (Δ*entB*) in-frame	This study
SAN180	Db10 (Δ*alb4-5*, Δ*entB*)	This study
SAN181	Db10 (Δ*alb4-5*, *swrA* : : Tn*5*, Δ*entB*)	This study
*Escherichia coli* MC1061	*F′lacIQ lacZM15* Tn*10* (Tet^R^), cloning host	Perego & Hoch (1988)
*E. coli* CC118λ*pir*	Cloning host and donor strain for pKNG101-derived marker exchange plasmids (λ*pir*)	Herrero *et al.* (1990)
*E. coli* HH26 pNJ5000	Mobilizing strain for conjugal transfer	Grinter (1983)
**Plasmids**		
pBluescript KS(+)	High copy cloning vector (Amp^R^)	Stratagene
pKNG101	Suicide vector for marker exchange (Sm^R^, *sacBR*, *mobRK2*, ori *R6K*)	Kaniga *et al.* (1991)
pSUPROM	Vector for constitutive expression of cloned genes under the control of the *E. coli tat* promoter (Kan^R^)	Jack *et al.* (2004)
pNW572	pKNG101-derived marker exchange plasmid for the generation of chromosomal Δ*alb4-5*	Gerc *et al.* (2012)
pSAN69	pKNG101-derived marker exchange plasmid for the generation of chromosomal Δ*entB*	This study
pSAN46	*pswP* coding sequence in pSUPROM	Gerc *et al.* (2012)

Amp^R^, ampicillin resistance; Kan^R^ , kanamycin resistance; Sm^R^, streptomycin ; Tet^R^, tetracycline resistance.

### Construction of strains and plasmids.

A *Ser. marcescens* chromosomal mutant with an in-frame deletion in *entB* was constructed by marker (allelic) exchange using the suicide vector pKNG101 (Kaniga *et al.*, 1991), as described previously (Coulthurst *et al.*, 2006). Briefly, the upstream and downstream flanking regions of the gene *SMA4415* (*entB*) were cloned into pBluescript KS(+) so as to generate a non-polar, in-frame deletion of the gene. This deletion allele was then cloned into pKNG101 and the resulting marker exchange plasmid introduced into *Ser. marcescens* by conjugation. Selection on streptomycin-containing agar and then on high sucrose agar allowed isolation of mutants in which the deletion allele had replaced the wild-type copy. The plasmids used in this study are detailed in Table 1. The upstream and downstream regions of *entB* were amplified using the primers AG157 (TATAAAGCTTTTTTGGAATGGCCATCGA) and AG158 (TATAGGGCCCACATCCTGACGACGCAAG) (upstream), and AG159 (TATAAAGCTTGAAGAGAAAGCCTGATTTTAATAATG) and AG160 (TATATCTAGATATTTGCCGGTGTCGGTC) (downstream). The resulting product was cloned into pBluescript KS(+) using restriction sites engineered into the primers (underlined in the primer sequences). Following marker exchange, the integrity of the disrupted region was confirmed by DNA sequencing. The Δ*alb4-5* Δ*entB* double mutant (SAN180) was constructed by introduction of the *entB* deletion into SAN5 (Δ*alb4-5*), as described above. The *swrA* : : Tn*5* mutation was introduced into the single Δ*alb4-5* mutant and the double Δ*alb4-5* Δ*entB* mutant by phage ϕIF3-mediated transduction as described previously (Petty *et al.*, 2006), generating strains SAN124 and SAN181, respectively. Growth curve measurement was also performed on each mutant strain, this showed that the growth rate was unaffected compared with wild-type *Ser. marcescens* Db10 (data not shown).

### Antimicrobial activity assays.

*Sta. aureus* was grown in liquid culture in a volume of 5 ml in a universal tube at 37 °C with rotation and 100 µl culture was spread onto an agar plate. Strains of *Ser. marcescens* being tested for antimicrobial production were grown in liquid culture in a volume of 5 ml in a universal tube at 30 °C with rotation and 10 µl culture was then spotted on the *Sta. aureus* lawn. The plates were incubated (15 h) at 30 °C prior to photography.

### CAS assay.

*Ser. marcescens* strains were grown to stationary phase at 30 °C and 10 µl of culture was spotted on to the CAS agar plate. The plates were incubated (15 h) at 30 °C prior to photography. For complementation analysis, plasmids were maintained by the addition of kanamycin to both the culture medium and to the CAS agar plates.

### Bioinformatic analysis.

The genome of *Ser. marcescens* strain Db11 was sequenced by the Pathogen Sequencing Unit, the Wellcome Trust Sanger Institute, Wellcome Trust Genome Campus, Hinxton, Cambridge, UK. *Ser. marcescens* strain Db11 is a spontaneous Sm-resistant mutant of *Ser. marcescens* strain Db10 and hence the Db11 genome information can be used for Db10. To identify NRPS or PKS secondary metabolite gene clusters encoded within the *Ser. marcescens* Db11 genome, the bioinformatics tool antiSMASH was employed (Blin *et al.*, 2013). The genome was uploaded as a nucleotide sequence and the bacterial, archaeal and plant plastid translational code, with a minimal gene length of 50, was used for gene finding by glimmer. Within antiSMASH, the following parameters were selected: smCOG analysis for functional prediction and phylogenetic analysis of genes, gene cluster blast analysis, subcluster blast analysis, whole genome pfam analysis and secondary metabolite detection on all possible ORFs. To investigate the likely function of NRPS or PKS products identified by antiSMASH, further manual analysis using blastp (Altschul *et al.*, 1997) was performed.

## Results and Discussion

### The PswP PPTase of *Ser. marcescens* Db10 is required for the biosynthesis of more than one metabolite with antimicrobial activity

**Fig. 1.**
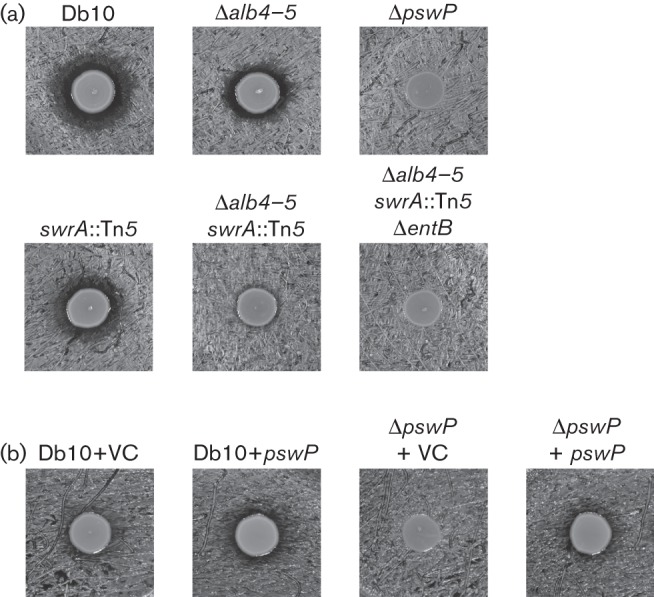
The PPTase PswP is required for the biosynthesis of three secondary metabolites with antimicrobial activity against *Sta. aureus*. (a) Antimicrobial activity assays using *Sta. aureus 113* as the indicator lawn. The producer strains are indicated above: Db10 (wild-type *Ser. marcescens* Db10), Δ*alb4-5* (SAN5), Δ*pswP* (SAN112), *swrA* : : Tn*5* (JESM267), Δ*alb4-5 swrA* : : Tn*5* (SAN124) and Δ*alb4-5 swrA* : : Tn*5* Δ*entB* (SAN181). (b) Antimicrobial activity assays show complementation of the *pswP* deletion by expression of the *pswP* gene *in trans*, using kanamycin-resistant *Sta. aureus* Newman *spa* : : tet *sbi* : : kan as the indicator lawn. The producer strains are indicated above: Db10+VC (*Ser. marcescens* Db10 pSUPROM, vector control), Db10+*pswP* (*Ser. marcescens* Db10 pSAN46), Δ*pswP*+VC (SAN112 pSUPROM) and Δ*pswP*+*pswP* (SAN112 pSAN46).

During a prior study investigating the mechanism of althiomycin biosynthesis in *Ser. marcescens* Db10, it was noted that while *Sta. aureus* was sensitive to althiomycin, disruption of althiomycin biosynthesis did not completely abolish the antimicrobial effect of *Ser. marcescens* Db10 against *Sta. aureus* (Gerc *et al.*, 2012). Aiming to investigate this further, we discovered that deletion of the gene encoding the PPTase protein PswP (which is required for althiomycin biosynthesis) was sufficient to negate the antimicrobial activity of *Ser. marcescens* Db10 against *Sta. aureus* (Fig. 1a). To confirm that the loss of antimicrobial activity associated with deletion of *pswP* was specific to deletion of this gene, the PswP mutant phenotype was complemented by expression of PswP *in trans*. Expression of PswP *in trans* restored the antimicrobial activity of *Ser. marcescens* Db10 against *Sta. aureus* (Fig. 1b). PPTase enzymes are known to modify the carrier protein domain of FAS, PKS and NRPS enzymes (Lambalot *et al.*, 1996). We hypothesized that, in addition to being required for althiomycin biosynthesis, PswP was required for the biosynthesis of one or more FAS, PKS or NRPS product(s) with antimicrobial activity against *Sta. aureus*. Following this initial observation, we aimed to determine the identity of this metabolite(s).

### Bioinformatic analysis of PswP and potential target proteins

**Fig. 2.**
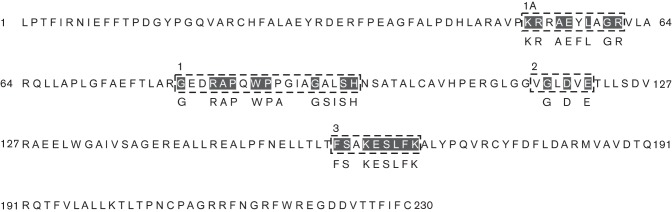
Identification of conserved motifs of the F/KES subfamily of Sfp-type PPTases within PswP of *Ser. marcescens* Db10. Conserved PPT motifs 1A, 1, 2 and 3 are denoted with a dashed boxed. Residues previously identified as being highly conserved across the F/KES subfamily are indicated below the amino acid sequence; residues of PswP that match the consensus shown are shaded black. Analysis is based on the motif alignment analysis performed by Copp & Neilan (2006).

PswP belongs to the Sfp class of PPTases and is one of three PPTases encoded by *Ser. marcescens* Db10 (Gerc *et al.*, 2012). There exists a second Sfp-type PPTase, SMA4147, which was previously identified during a search for the PPTase required for the biosynthesis of althiomycin; however, no biological function for SMA4147 has been ascribed (Gerc *et al.*, 2012). SMA3052 represents a third PPTase encoded by *Ser. marcescens* Db10 that shows homology to AcpS family PPTases (data not shown) and is therefore likely involved in primary metabolism (Lambalot *et al.*, 1996). Therefore, further bioinformatic analysis of the PPTases encoded by *Ser. marcescens* Db10 focused solely on PswP. Based on the presence of the conserved residues within motifs 1A, 1, 2 and 3, we assigned PswP to the F/KES subfamily of Sfp-type PPTases (Fig. 2).

In *Ser. marcescens* Db10, the *pswP* gene is not embedded within or adjacent to an NRPS or PKS biosynthetic gene cluster. We were therefore unable to predict additional PKS or NRPS biosynthetic pathways that might require the activity of PswP based on its genomic context. The bioinformatics tool, antiSMASH, allows the identification and the preliminary analysis of secondary metabolite gene clusters in bacterial and fungal genomes to be performed in an unbiased manner (Blin *et al.*, 2013). Therefore, antiSMASH was employed to identify all genomic regions containing putative PKS, NRPS and NRPS-PKS gene clusters in the *Ser. marcescens* Db10 genome, on the basis that one or more of these might direct the biosynthesis of the secondary metabolite(s) active against *Sta. aureus*. The results of this search are summarized in Table 2. Five genomic regions containing NRPS or NRPS-PKS gene clusters, whose products could potentially be modified by the action of PswP, were identified. Upon closer inspection of the NRPS and NRPS-PKS gene clusters, the althiomycin biosynthetic machinery and the machinery required for the biosynthesis of the surfactant, serrawettin W2, were identified in clusters three and four (Gerc *et al.*, 2012; Pradel *et al.*, 2007). Published data for the three remaining gene clusters was not available; therefore, a bioinformatic approach was adopted. In an attempt to deduce the likely product of the three remaining NRPS biosynthetic gene clusters, the protein sequences encoded by the NRPS genes(s) identified by antiSMASH and the products of their flanking genes were subjected to database searching using blastp (Altschul *et al.*, 1997). Analysis of the NRPSs in gene cluster one revealed that this locus likely encodes proteins involved in the biosynthesis of a microcin (Table 2). SMA1574A shows 41 % identity with microcin N of *E. coli* 2424 (Corsini *et al.*, 2010) and the NRPS proteins, SMA1572 and SMA1571, are likely involved in the biosynthesis of a modifying group added to the microcin peptide encoded by this gene cluster (Rebuffat, 2012). In contrast, gene clusters two and five appear to direct the synthesis of multiple siderophores (Table 2). The NRPS encoded within gene cluster two shares homology with chrysobactin synthetase component F (Persmark *et al.*, 1989). Within gene cluster five is a locus containing an NRPS and associated genes conserved with the enterobactin biosynthesis and utilization genes from *E. coli* (see below) (Raymond *et al.*, 2003).

**Table 2.** Characterization of NRPS- or PKS-encoding genes identified by antiSMASH (Blin *et al.*, 2013)

Gene cluster	Genomic region predicted by antiSMASH	Enzyme predicted by antiSMASH	Gene(s) encoding NRPS (or NRPS-PKS)	Previously reported product	Putative product	Comments
1	*SMA1552–1589*	NRPS	*SMA1571-1572*	–	Unknown	*
2	*SMA1705–1744*	NRPS	*SMA1729*	–	Siderophore	†
3	*SMA2274-1309*	NRPS-PKS	*SMA2289-2290*	Althiomycin	na	‡
4	*SMA3659–3702*	NRPS	*SMA3680*	Serrawettin W2	na	§
5	*SMA4386–4438*	NRPS	*SMA4411*	–	Siderophore	||
*SMA4402-4404, SMA4406*	–	Unknown	¶

na, not applicable.

* Predicted NRPS encoded within a cluster of genes (*SMA1575–1568*) showing similarity with genes involved in the biosynthesis and secretion of microcins; may synthesize a modifying group added to the microcin.

† Predicted NRPS similar to chrysobactin synthetase (64 % identity over the whole length) and flanked by genes encoding proteins with similarity to proteins mediating siderophore export, uptake and iron release.

‡ Althiomycin is a broad-spectrum antibiotic. NRPS-PKS is encoded within the six-gene *alb* operon (*SMA2288–2293*), which also encodes tailoring and export functions (Gerc *et al.*, 2012).

§ Serrawettin W2 is a biosurfactant (Pradel *et al.*, 2007).

||Predicted NRPS is similar to enterobactin synthetase EntF and within cluster of genes (*SMA4408–4415*) similar to the enterobactin biosynthesis, export and uptake cluster of *E. coli* (Fig. 3).

¶ Encoded NRPS proteins share similarity with siderophore synthetase enzymes from other organisms but number and nature of product(s) unclear.

### The PswP-dependent surfactant serrawettin W2 has antimicrobial activity against *Sta. aureus*

Biosurfactants are amphipathic molecules whose primary function is to reduce surface tension to allow bacterial spreading across surfaces (Matsuyama *et al*., 2011). Biosurfactants have been shown to possess several properties, including antimicrobial activity (Singh & Cameotra, 2004). Of particular note, serrawettin W1 produced by *Ser. marcescens* ATCC 274 has been shown to have antimicrobial properties against meticillin-resistant *Sta. aureus* (MRSA) (Kadouri & Shanks, 2013). In *Ser. marcescens* Db10, PswP was previously shown to be required for the biosynthesis of serrawettin W2 (Pradel *et al.*, 2007). Therefore, to determine whether serrawettin W2 also possessed antimicrobial activity against *Sta. aureus*, a serrawettin W2 single mutant was obtained (Pradel *et al.*, 2007) and used to construct a double mutant (Δ*alb4-5 swrA* : : Tn*5*) that was unable to produce althiomycin or serrawettin W2. Both mutant strains were tested for antimicrobial activity against *Sta. aureus* (Fig. 1a). Compared with *Ser. marcescens* Db10, the *swrA* : : Tn*5* mutant showed a slight reduction in antimicrobial activity against *Sta. aureus*. The reduction in size of the antibiosis halo observed with the double mutant (Δ*alb4-5 swrA* : : Tn*5*) was much more pronounced compared with either single mutant; however, a small antibiosis halo could still be observed (Fig. 1a). Therefore, we concluded that serrawettin W2 does indeed possess antimicrobial activity against *Sta. aureus*, but that *Ser. marcescens* Db10 must produce another antimicrobial, in addition to serrawettin W2 and althiomycin, which is dependent on the presence of PswP for biosynthesis.

### The *SMA4408-4415* gene cluster encodes a siderophore that is dependent on PswP for biosynthesis and has antimicrobial activity against *Sta. aureus*

**Fig. 3.**
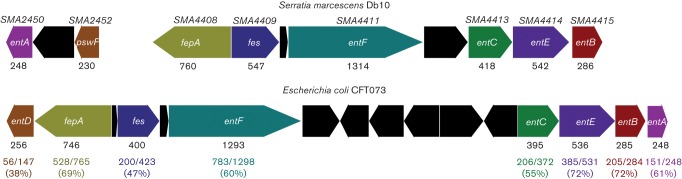
Comparison of the *SMA2450–2452* and *SMA4408–4415* gene clusters of *Ser. marcescens* Db10 with the enterobactin biosynthetic gene cluster of *E. coli* CFT073 (*c0668–c0683*). Genes are drawn approximately to scale and protein length is indicated below the encoding gene (as the number of amino acids). Homologous genes are indicated by the same colour, the level of identity between homologous proteins is shown below the corresponding genes as an absolute value and as a percentage.

**Fig. 4.**
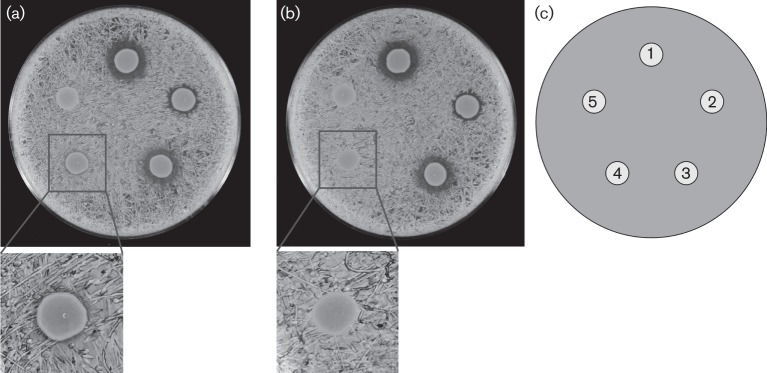
An althiomycin and serrawettin W2 mutant of *Ser. marcescens* Db10 is unable to kill *Sta. aureus* in the presence of additional iron. Antimicrobial activity assays in the absence (a) or presence (b) of 50 μM FeCl_3_, using *Sta. aureus* as the indicator lawn, with a schematic of the location of each producer strain shown (c). The producing strains were: 1, *Ser. marcescens* Db10 (wild-type); 2, SAN5 (Δ*alb4-5*); 3, JESM267 (*swrA* : : Tn*5*); 4, SAN124 (Δ*alb4-5 swrA* : : Tn*5*); 5, SAN112 (Δ*pswP*). The insets show a magnified image of producer strain 4 SAN124 in both (a) and (b).

In light of the results presented above, we reasoned that one or more of the three remaining NRPS gene clusters identified by antiSMASH should be responsible for the antimicrobial effect against *Sta. aureus* observed in the absence of althiomycin and serrawettin W2. Two of the three remaining NRPS gene clusters, identified by antiSMASH, encoded proteins with similarity to siderophore biosynthetic proteins (Table 2, Fig. 3). We therefore hypothesized that either a siderophore produced by *Ser. marcescens* Db10 was directly toxic toward *Sta. aureus*, or that siderophore-dependent removal of iron from the environment prevented growth of *Sta. aureus*. In order to investigate these possibilities, FeCl_3_ was added to the growth media and the antimicrobial bioassay repeated. The small antibiosis halo observed in the absence of additional iron with the double Δ*alb4-5 swrA* : : Tn*5* mutant disappeared upon the addition of FeCl_3_ (Fig. 4). From these results two scenarios are possible: 1) addition of excess iron negates the iron depletion effect of a siderophore(s), or 2) the addition of iron negatively affects the production of siderophores by *Ser. marcescens* Db10, as observed in *E. coli* and many other Gram-negative bacteria (Crosa & Walsh, 2002). In either case, these findings indicated that siderophore production by *Ser. marcescens* Db10 was likely to inhibit *Sta. aureus* growth.

**Fig. 5.**
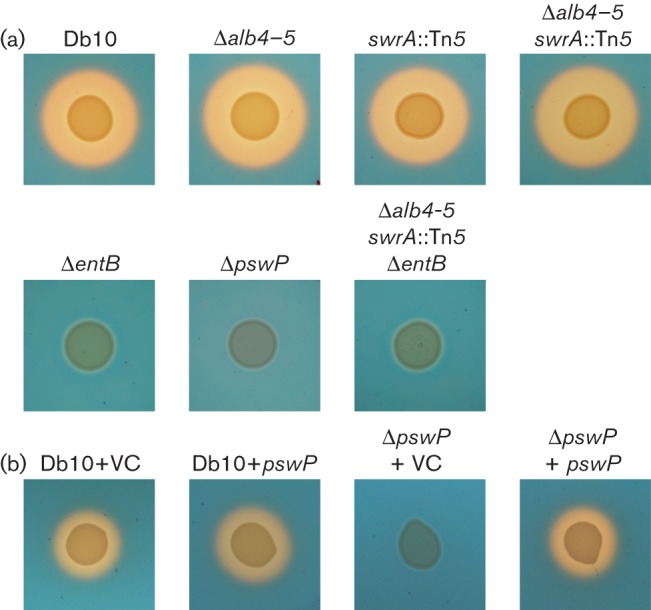
The PPTase PswP and SMA4415 (EntB) are required for the biosynthesis of a siderophore in *Ser. marcescens* Db10. CAS assay for the detection of siderophore biosynthesis, as indicated by the presence of an orange halo. (a) The producer strains are indicated above: Db10 (wild-type *Ser. marcescens* Db10), Δ*alb4-5* (SAN5), *swrA* : : Tn*5* (JESM267), Δ*alb4-5 swrA* : : Tn*5* (SAN124), Δ*entB* (SAN176), Δ*pswP* (SAN112) and Δ*alb4-5 swrA* : : Tn*5* Δ*entB* (SAN181). (b) Complementation of the *pswP* deletion by expression of the *pswP* gene *in trans*. The producer strains are indicated above: Db10+VC (*Ser. marcescens* Db10 pSUPROM, vector control), Db10+*pswP* (*Ser. marcescens* Db10 pSAN46), Δ*pswP*+VC (SAN112 pSUPROM) and Δ*pswP*+*pswP* (SAN112 pSAN46).

As mentioned above, a set of genes (*SMA4408–4415*) within cluster five show homology and synteny with the enterobactin gene cluster of *E. coli* (Fig. 3). It therefore seemed likely that these genes are involved in the synthesis of enterobactin or a closely related molecule. However, it should be noted that the only close homologue of *entA*, which is located immediately upstream of *entB* in *E. coli*, was identified as *SMA2450* in *Ser. marcescens* Db11. In contrast with *E. coli*, the *SMA2450* gene is located at a distant locus from the other enterobactin-like genes; however, it is closely genetically linked with *pswP* (*SMA2452*) in *Ser. marcescens*. Given both the linkage between *entA* and *pswP* in *Ser. marcescens* and that the *SMA4408–4415* genomic region showed the most convincing similarity with a well-characterized cluster of genes involved in siderophore biosynthesis and utilization, we tested whether the product of these genes was responsible for the PswP- and iron-dependent growth inhibition observed in the Δ*alb4-5 swrA* : : Tn*5* double mutant. The product of *SMA4415* shares significant homology with *entB* of the enterobactin biosynthetic gene cluster (Fig. 3). Since EntB is essential for enterobactin biosynthesis in *E. coli* (Crosa & Walsh, 2002), we reasoned that SMA4415 would be essential for biosynthesis of this siderophore in *Ser. marcescens* Db10. An in-frame deletion in *entB* was therefore constructed in *Ser. marcescens* Db10, and also in the althiomycin and serrawettin W2 mutant background to give a triple althiomycin, serrawettin W2 and Δ*entB* mutant (Δ*alb4-5 swrA* : : Tn*5* Δ*entB*). To compare siderophore production by wild-type *Ser. marcescens* Db10 with that of the single Δ*entB* mutant, the triple Δ*alb4-5 swrA* : : Tn*5* Δ*entB* mutant and the Δ*pswP* mutant, the relevant strains were grown on CAS indicator plates. CAS/hexadecyltrimethylammonium bromide complexed with ferric iron present in these plates serves as an indicator of siderophore biosynthesis; in the presence of an iron chelator this indicator turns from a blue to an orange colour (Schwyn & Neilands, 1987). In wild-type *Ser. marcescens* Db10, production of a diffusible siderophore was clearly observed (Fig. 5a). In contrast, no siderophore production was observed in either the Δ*entB* or PswP PPTase (Δ*pswP*) single mutants (Fig. 5a). Furthermore, no siderophore production was observed in the althiomycin, serrawettin W2 and Δ*entB* triple mutant (Δ*alb4-5 swrA* : : Tn*5* Δ*entB*) (Fig. 5a). The loss of siderophore production was specific to the deletion of *entB* or *pswP*, with siderophore production being unaffected in strains unable to produce althiomycin and/or serrawettin (Fig. 5a) These results clearly show that both *entB* and *pswP* are essential for the biosynthesis of a siderophore in *Ser. marcescens* Db10. In addition, this assay confirmed that construction of the *pswP* (*SMA2452*) PPTase mutant did not affect siderophore biosynthesis through downstream polar effects on the expression of *SMA2450*, which is predicted to encode an EntA homologue (Fig. 3), since the loss of siderophore biosynthesis observed with the *pswP* mutant could be complemented by the expression of *pswP in trans* (Fig. 5b). Having shown that *entB* was required for biosynthesis of a siderophore in *Ser. marcescens* Db10, the triple althiomycin, serrawettin W2 and siderophore mutant was tested for antimicrobial activity against *Sta. aureus*. No antibiosis halo was observed with the Δ*alb4-5 swrA* : : Tn*5* Δ*entB* triple mutant (Fig. 1a), thus providing a phenocopy of the PswP PPTase mutant and identifying the *entB*-dependent siderophore as the third PswP-dependent antimicrobial secondary metabolite produced by *Ser. marcescens* Db10.

## Concluding remarks

In this study, we have shown that *Ser. marcescens* Db10 produces three diffusible molecules with antimicrobial activity against the clinically relevant pathogen *Sta. aureus*. Production of all three requires PswP, consistent with their biosynthesis being dependent on NRPS enzymes. Genetic evidence has revealed these three metabolites to be: the antibiotic althiomycin, the product of Alb1-6 (Gerc *et al.*, 2012); the biosurfactant serrawettin W2, the product of SwrA (Pradel *et al.*, 2007); and a siderophore whose production is associated with NRPS-containing cluster of genes, *SMA4408–4415*. Importantly, this is the first time, to our knowledge, that the latter two molecules have been shown to act against *Sta. aureus*. Furthermore, production of this siderophore and its association with PswP and the *SMA4408–4415* gene cluster has not been reported previously in *Ser. marcescens*. Whilst we speculate that this molecule is likely to be closely related, or even identical, to enterobactin produced by *E. coli*, its chemical structure remains to be confirmed. Whilst we speculate that the ability to use this siderophore to scavenge Fe^3+^ is likely to be important for *Ser. marcescens* to survive within the iron-limited environment of the host during infection (Miethke & Marahiel, 2007), this remains to be investigated. In addition, our data highlight how an individual bacterial PPTase enzyme can play an essential role in the biosynthesis of multiple secondary metabolites with disparate physiological roles. PswP provides another example, in addition to others reported previously (Beld *et al.*, 2014), illustrating that PPTase enzymes are not necessarily pathway-specific or genetically linked with their substrates but may act on multiple NRPS enzymes. Finally, our data also illustrate how several distinct secondary metabolites can contribute to the inhibition of growth of competitor bacteria, a scenario that is likely to occur at polymicrobial infection sites and in varied environmental niches. In particular, we note the potential utility of iron restriction to inhibit the growth of clinically significant pathogens such as *Sta. aureus*.

### Note added in proof

The siderophore product dependent on the enterobactin-like gene cluster *SMA4408-4415* is likely to be identical or related to the serratiochelin molecules recently reported to be produced by *Serratia* sp. V5 using a ‘shuffled’ combination of enterobactin-like and vibriobactin-like genes (Seyedsayamdost *et al.*, 2012).
